# Evaluation of COVID-19 pandemic management in Türkiye

**DOI:** 10.3389/fpubh.2023.1142471

**Published:** 2023-03-23

**Authors:** Umit Kartoglu, Kayihan Pala

**Affiliations:** ^1^Extensio et Progressio, Collonge-Bellerive, Switzerland; ^2^Department of Public Health, Faculty of Medicine, Bursa Uludag University, Bursa, Türkiye

**Keywords:** pandemic management, prevention, preparedness, readiness, response, recovery, resilience, COVID-19

## Abstract

Three years since the first cases were identified and 2 years since an effective vaccine was developed, COVID-19 continues to claim lives and impact people's health and wellbeing, both socially and economically. While the world has been waiting for its leaders to come together to form a collective response to end the pandemic, we still have not seen a multisectoral response, nor any whole-of-society approach. Like many other countries around the globe, Türkiye was caught unprepared by the pandemic. This was exacerbated by the unsuccessful management of the pandemic by the authorities. The reasoning and/or scientific explanations for enforcing or lifting public health measures have never shared with the public. Throughout the pandemic, no epidemiological details have been released on cases and deaths, other than the numbers of these two measures. Civil society organizations, professional associations, and the public in general have been kept out from policy formulation and decision making. As a result, community engagement has never been properly put into practice. In this paper, we analyzed Türkiye's pandemic management response through the continuum of the response cycle to emergencies: prevention, preparedness, readiness, response, and recovery.

## Introduction

COVID-19 continues to amass many cases, impact physical and mental health, and claim lives. It is marked as the most significant public health emergency in over a century. To date, a total of more than 600 million cases and 6.5 million deaths have been reported (1).

Despite warnings of potential pandemics through specialized agencies and various initiatives, the world was caught off guard by COVID-19.

In its *2007 World Health Report*, the World Health Organization (WHO) called for global solidarity to achieve better security against universal vulnerability, including outbreaks of emerging and epidemic-prone diseases and successful implementation of International Health Regulations (2). In May 2017, Time magazine brought a similar topic to its cover—*Warning: We are not ready for the next pandemi*c (3). This was done in the middle of the 5^th^ and the biggest epidemic of the H7N9 virus that began in October 2016 (4). The World Health Organization, in its 2018 publication *Managing epidemics*, underlined the following for a future pandemic: “with a high degree of certainty, […] when it comes, there will be (a) an initial delay in recognizing it; (b) a serious impact on travel and trade; (c) a public reaction that includes anxiety, or even panic and confusion, and (d) this will be aided and abetted by media coverage (5).” In September 2019, just 3 months before the coronavirus outbreak sparked in China, the Global Preparedness Monitoring Board (GPMB) released its first annual report, *A World at Risk*. This provided a snapshot of the world's ability to prevent and contain a serious global threat, and seven urgent priority actions leaders must take in the face of said threat (6). Similarly, *The Global Health Security Index 2019* report was released just two months before the first COVID-19 cases (7). However, its findings on pandemic preparedness did not match what we have observed since the emergence of COVID-19. Countries who experienced SARS, although not top scorers in the index (e.g., Vietnam), demonstrated an exemplary fight against the pandemic.

In 1999, WHO published a global influenza preparedness plan, urging countries to develop and update national plans according to the recommendations in the guideline (8). The guidance document was updated in 2005 (9). However, following the H1N1 pandemic, it was found that only a limited number of countries had updated their plans (10). These revised national plans were critical and the only available tool in hand when the COVID-19 pandemic emerged. WHO recommended the development of country-specific operational plans for COVID-19 preparedness and response, or an adaptation of the existing Influenza Pandemic Preparedness Plan (11).

With the COVID-19 pandemic, countries were focused on preparing and responding to the disease. Although these steps were critical, response to the disease is just one element in a continuum of a response cycle to emergencies as indicated by WHO: “Prevention, preparedness, readiness, response, and recovery lie on a continuum and to be effective, this continuum needs comprehensive attention (12).” In this response cycle, the steps overlap but also have their own peculiarities. In this paper, we analyzed Türkiye's pandemic management under the elements of this continuum.

**Figure 1:**
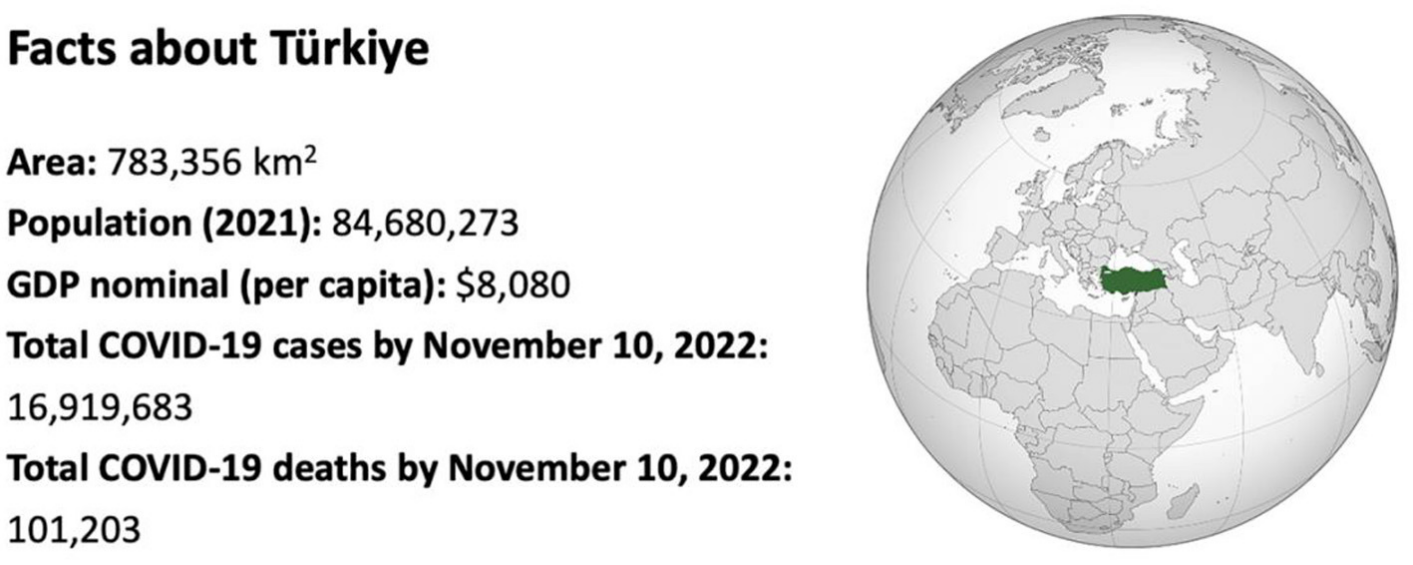
Facts about Türkiye.

Facts about Türkiye is given in Figure 1.

## Prevention

The world's initial reaction to cases from Wuhan, China and the WHO's declaration of a pandemic was the closure of borders, as well as restrictions on domestic travel in many countries. Restricting the movement of people can be useful at the early stages of an outbreak to gain some time and implement effective preparedness measures (13). A modeling study found that in May 2020, imported cases were likely to have accounted for more than 10% of total COVID-19 cases in 102 countries, but dropped significantly by September (14). Despite other modeling studies finding similar results on the minimal impact of border closures on the spread of COVID-19, many countries enforced new travel bans following the emergence of the Omicron variant from South Africa, which was called out as discriminatory by Africa's leaders (15).

Along with many other countries, Türkiye also introduced an international travel ban starting with China, then expanding to other affected areas, before ultimately fully shutting down its borders on March 31, 2020 (16). When travel was allowed, the main detection method was thermal passenger screening for COVID-19 infection at airport exit and entry which would not detect half of the infected travelers (17).

From a risk management perspective, a control measure will always have flaws and will be short-lived if not combined with other effective control measures such as testing, contact tracing, isolation of cases, and quarantining, in addition to personal preventive measures such as wearing face masks, regular hand washing, and proper cough hygiene.

## Preparedness and readiness

With infectious disease outbreaks becoming a growing threat, it became necessary to carry out intensive studies on pandemic preparations globally. WHO published the 3rd edition of the International Health Regulations (IHR) in 2005 on the control of epidemics, public health emergencies, and pandemics. The IHR is a binding regulation for the 194 Member States of the World Health Organization and two additional countries/territories. This agreement defines the criteria for public health emergencies of international importance and regulates the obligations of countries should such a situation occur (18).

The Turkish Ministry of Health published the *Pandemic Influenza National Preparedness Plan* in 2006. It was last updated in October 2019 in light of the lessons learned from the 2009 H1N1 pandemic (19). Although COVID-19 is not caused by the influenza virus, the main framework of influenza preparedness also constitutes the main framework of the COVID-19 pandemic response. However, developments during the early stages of the pandemic raise questions about the extent to which this main framework has been implemented during the entirety of the pandemic. The fact that the *Pandemic Influenza National Pandemic Preparedness Plan* was updated in Türkiye just before the COVID-19 pandemic was an important opportunity to give a strong response to the current pandemic. Unfortunately, no study has been conducted in Türkiye on the rapid adaptation of this plan to COVID-19 (20).

The Technical Committee for the influenza pandemic was converted into a wider group on January 10, 2020, the Scientific Advisory Committee, much earlier than in many other countries. However, the Committee had only one epidemiologist among its infectious diseases, microbiology, virology, internal medicine, intensive care, and pulmonology specialists. By April 2020, the number of members in the Committee reached 38, with some additional epidemiologists, though not all members were invited to every meeting. Although there was a recommendation for a Social Sciences Advisory Committee to be established, this never took place. A side committee was formed with the involvement of social scientists, but this committee had a very low profile and no impact on the management of the pandemic. The National Coordination Board, that has the responsibility of coordinating ministries and institutions during outbreaks, has not been activated at all during the pandemic. It can be concluded that, as Türkiye did not have an epidemic strategy and preparedness plan adapted to the COVID-19 pandemic, it is debatable to what extent the current pandemic preparedness plan has been implemented. Despite the Scientific Advisory Committee advising the Ministry of Health to issue the announcements on lockdowns and measures, these activities were carried out by the Ministry of Interior (21).

In an evaluation based on the types of roles of scientists as defined by Pielke, the Scientific Advisory Committee in Türkiye was not considered to possess the two ideal types of roles for decision-making committees—the “pure scientist” (focused only on the scientific reality, not involved in politics and they stay away from decision making processes) and the “science arbiter” (stay away from politics and decision making processes, respond to decision-makers' requests on specific issues and give scientific advice, and may conduct studies on the issues requested). It was concluded that the members of the Committee see themselves as “honest brokers” (in this intermediate role, scientists produce alternative preferences for selection as much as possible for decision making along with political trend), but they more so correspond to the remaining role of the “issue advocate” (involved in political processes and decision making processes and work for supporting the objectives defined by the political agenda), since they are involved in a political process (22).

## Response

WHO stated that preparation and strategy plans for the COVID-19 pandemic should be based on the influenza pandemic preparation plans and prepared guidelines in the early period of the pandemic (11). WHO set six strategic objectives to respond to the COVID-19 pandemic (Box 1) (23).

Box 1 **Six strategic objectives to respond to COVID-19 pandemic (WHO).**

• Suppress transmission through the implementation of effective and evidence-based public health and social measures, and infection prevention and control measures, including detecting and testing suspected cases; investigating clusters of cases; tracing contacts; supported quarantine of contacts; isolating probable and confirmed cases; measures to protect high-risk groups; and vaccination.
• Reduce exposure by enabling communities to adopt risk-reducing behaviors and practice infection prevention and control, including avoiding crowds and maintaining physical distance from others; practicing proper hand hygiene; through the use of masks; and improving indoor ventilation.
• Counter misinformation and disinformation by building resilience through managing the infodemic, communicating with, engaging, and empowering communities, enriching the information eco-system online and offline through high-quality health guidance, and by communicate risk and distilling science in a way that is accessible and appropriate to every community.
• Protect the vulnerable through vaccination, ensuring vaccine deployment readiness in all countries and all populations, by communicating, implementing, and monitoring COVID-19 vaccination campaigns, by engaging health workers, and by building vaccine acceptance and demand based on priority groups, taking into account gender and equity perspectives to leave no one behind.
• Reduce mortality and morbidity from all causes by ensuring that patients with COVID-19 are diagnosed early and given quality care; that health systems can surge to maintain and meet the increasing demand for both COVID-19 care and other essential health services; that core health systems are strengthened; that demand-side barriers to care are addressed; and by ensuring that all priority groups in every country are vaccinated.
• Accelerate equitable access to new COVID-19 tools including vaccines, diagnostics and therapeutics, and support safe and rational allocation and implementation in all countries.

### Detection of cases and treatment

#### Standard case definitions and notifications

The WHO shared the standard case definition for COVID-19 in a document published on March 25, 2020 and recommended two different international codes for case and death records: U07.1 (COVID-19, cases with defined virus, confirmed by laboratory test) and U07.2 (COVID-19, unidentified virus, clinical-epidemiological diagnoses, probable cases and suspected cases) (24). On April 16, 2020, the WHO also published a guide on international rules for certification and classification in cases where COVID-19 was the cause of death (25). However, the Ministry of Health in Türkiye only publicized confirmed cases and confirmed deaths in which SARS-CoV-2 was detected by molecular methods. In Türkiye, no data was disclosed regarding clinically and epidemiologically diagnosed cases, probable/suspected cases, and those who lost their lives. Furthermore, only the total numbers of confirmed cases and deaths were disclosed, without any details such as age, gender, occupation, social class, province of residence, and concomitant disease (26).

#### Testing policy

WHO recommends that all suspected cases be tested for COVID-19 according to the Organization's case definitions. WHO emphasizes that all countries must increase their level of preparedness, alertness, and response to identify, manage, and deal with new cases of COVID-19, and that laboratory testing is an integral part of this strategy (27). Türkiye's testing policy has been adopted as testing only those with symptoms. During the pandemic, it has been recommended to carry out more than 5 tests per thousand people per day in order to monitor the course of the epidemic (28). Unfortunately, Türkiye never reached 5 tests per thousand people per day in 2020 and 2021, and no test figures are available for 2022. In the first 6 months of the pandemic in Türkiye, very few tests were carried out— <1 per thousand people (29). Rapid antigen tests have never been made available/implemented.

#### Treatment protocols (guidelines)

In the first days of the pandemic in Türkiye, hydroxychloroquine was put into use by the Ministry of Health. Despite the Turkish Medical Association and many other medical specialty associations calling on the Ministry of Health to remove hydroxychloroquine from treatment plans due to a lack of scientific evidence of its benefits, it took one full year for the Ministry to do so in May 2021 (30–32).

The Ministry of Health added favipiravir in addition to hydroxychloroquine to its COVID-19 treatment guideline in July 2020. Medical specialty associations emphasized that the results from ongoing randomized controlled clinical trials on favipiravir should be closely monitored and requested that results from scientific studies from Türkiye be carefully evaluated. However, as with hydroxychloroquine, the Ministry of Health did not disclose any reports regarding the use of favipiravir. Publications showed that the effect of favipiravir could not be proven and was indeed considered ineffective in the treatment of COVID-19 as early as September 2020 (33–35). Nevertheless, the Ministry of Health did not immediately consider the results of these studies. Favipiravir was removed from the treatment guideline in December 2021, only after approximately one and a half year of use.

### Risk mitigation strategies

The pandemic requires robust risk control strategies that are multi-layered, science-based, and subjected to effectiveness checks and formal reviews (36). There are no signs of use of the risk management approach in Türkiye's response in control and mitigation strategies of COVID-19. Decisions taken in the country like the introduction or loosening of non-pharmaceutical interventions, as well as testing, did not have a scientific basis. For example, on May 22, 2022, the Minister of Health announced that masks would no longer be obligatory when the daily cases stayed under 1,000 over three consecutive days (37). Following this, the Ministry of Health started to share only weekly numbers of cases, and no change in policy occurred despite average daily cases reached over 1,000 within a week, and over 33,000 cases during the first week of August 2022 (38). In addition, the Ministry continues to lag in releasing weekly statistics.

#### Active surveillance system

Active surveillance is the ongoing systematic collection, analysis, and interpretation of data through patient screening when an outbreak of an infectious disease occurs or is expected to begin. This useful evidence, when provided timely, helps decision makers in leading and managing outbreaks more effectively. It also helps in directly measuring the effects of interventions (39). During the COVID-19 pandemic, an active surveillance system was not established in Türkiye. In addition, some approaches were found to be harmful to the control of the situation, i.e., the Ministry recommending no measures to be taken for close contacts when a possible COVID-19 case is detected until laboratory results are obtained (40).

#### Non-pharmaceutical interventions

##### Personal preventive measures (face masks, hand hygiene, and proper etiquette when sneezing and coughing)

Face masks play an important role in the prevention of COVID-19. With the announcement of the first confirmed case by the Ministry of Health on March 11, 2020, mask prices in Türkiye multiplied 25 times. At the beginning of the pandemic, the Government painted a disorganized picture regarding mask distribution. First, it was said that masks would be sold in markets. Following public outrage, the sale of the masks was completely banned, and it was announced that they would be distributed free of charge. However, it turned out that the Government did not prepare any plan to implement this. During the month following the free mask distribution announcement, the Government changed distribution channels several times. Free mask distribution was unsuccessful, ending with masks ultimately being sold in markets as originally announced (41). Health facilities and Family Health Centers especially experienced serious shortages of protective equipment, e.g., masks, aprons, gloves, and disinfectants. Health personnel had to approach the market, with the prices of such equipment having sharply increased (20, 42).

##### Physical distancing (contact tracing, isolation of patients, quarantine, school measures and closings, workplace measures and closings, avoiding crowded places, and working from home)

Although the trajectory of the outbreak was clear by the end of January 2020, it took months for the Ministry of Health of Türkiye to organize contact tracing teams. Some teams were composed of health workers with no training and non-health professionals. In the end, contact tracing turned into an activity used solely to locate contacts and send them medicine. Moreover, contacts were not routinely tested, being asked to sign a consent form and quarantine themselves at home for 14 days (43).

Pandemic clinics were opened for those hospitalized, and medical isolation measures were taken. However, no isolation arrangements were made for confirmed cases with no hospitalization indication. These patients were expected to isolate themselves at home. Hospitals were largely unprepared for the pandemic process. Starting from the very first case in the country, all hospitals faced logistical problems and lack of personal protective equipment (PPE) (44). A Turkish Medical Association (TMA) report on the 6th month of the pandemic details the problems observed in public hospitals during the pandemic (45).

The very late initiation of filiation in Türkiye led to a rapid spread of the disease with the basic reproduction number (R0) hitting 9.6 as announced by the Ministry of Health on Day 10 of the epidemic (46). This was much greater than the highest R0 (6.5) that was estimated in a systematic review (47).

In Türkiye, after the report of the first case on March 11, 2020, face-to-face education was suspended five days later, with the recommendation of distanced education at all levels. Schools were reopened gradually in September 2020, but with the increasing number of cases, many educational institutions switched back to online courses for the fall semester of 2021. No active or passive surveillance was conducted to monitor the cases and deaths of students, teachers, and families at risk at home (especially the 60+ age group) during the open and closed periods of schools.

Although the government made some arrangements regarding intermittent attendance for those working in the public sector at the beginning of the pandemic, no such arrangements were made regarding the closings for those working in the private sector. On the contrary, as in the example of a food production plant in Çanakkale province, upon the detection of SARS-CoV-2 among its employees, the workers were quarantined for 14 days in the plant in order to continue working with the decision of the Çanakkale Governorate Provincial Public Health Board (48). Forcing a confirmed case to work was not only hazardous for employee health, but also against any legal regulation.

According to the report of the Occupational Health and Safety Council, at least 1,400 workers died in the second year of the pandemic due to COVID-19. COVID-19 deaths were mostly recorded among health workers, education workers, office workers, municipal workers, security guards, and factory workers—especially in the metal and textile sectors (49).

There were many partisan approaches and decisions regarding lockdowns and the banning or limiting the number of attendants to public gatherings. While public gatherings were limited to 300 participants in March 2021, no sanctions were brought to ruling party congresses that brought over 1,000 people together (50).

##### Travel related measures (travel advice, screening of arriving passengers, travel limitations, and border closing)

The SARS CoV-2 virus isolated in Türkiye in April 2020 was predominantly of Saudi Arabian and Iranian origin. During the early days of the pandemic, the uncontrolled entry of people returning from Umrah, Saudi Arabia, to Türkiye and the delays in controlling transit from Iran to the country had a major impact on the spread of the disease (20).

Türkiye could not prepare and put into effect an effective action plan on the monitoring and control of in-country transportation. So much so that after the increase in the number of confirmed cases in Istanbul, which was called the Wuhan of Türkiye by the Ministry of Health, there was no restriction nor any control on the transition to Anatolia. A few weeks later, and during the summer holidays especially, the number of cases increased sharply in the provinces of Anatolia. This was called “COVID's migration” (51).

#### Pharmacological interventions (vaccination)

On November 25, 2020, the Minister of Health announced that an agreement had been reached with Sinovac on the supply of 10 million doses (52). The first shipment of the vaccine arrived in Türkiye on December 30, 2020 (53). Following the issuance of emergency use authorization, the vaccination campaign started on January 13, 2021 (54). When questioned why the Ministry did not consider mRNA vaccines, the Minister claimed that inactive vaccines are the most trusted vaccines as we do not know mRNA vaccines' long-term effects (55). Despite this claim, the Ministry introduced the Pfizer-BioNTech vaccine into the program on April 12, 2021. The Minister's earlier criticism on the unknown effects of mRNA vaccines was picked up by conspiracy theorists and anti-vaccine groups and was used heavily against the vaccination program. In April 2021, the Ministry gave emergency use authorization to Sputnik V. The first party of vaccines arrived in Türkiye in June 2021 but was never introduced for use in the program (56). The Ministry of Health never responded to inquiries to this effect.

**Figure 2:**
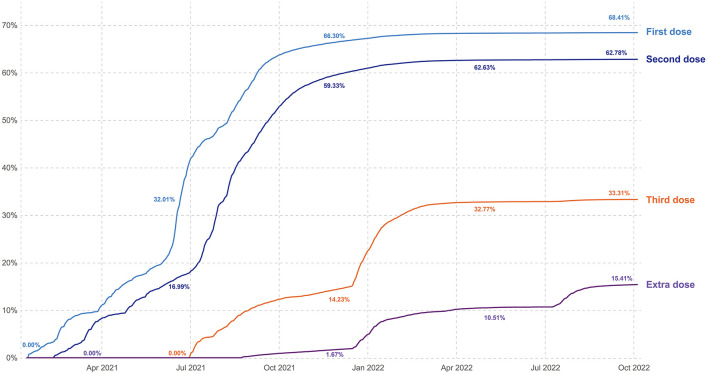
Cumulative proportion of total population vaccinated by time (graphic Zeki Berk, Türkiye).

**Figure 3:**
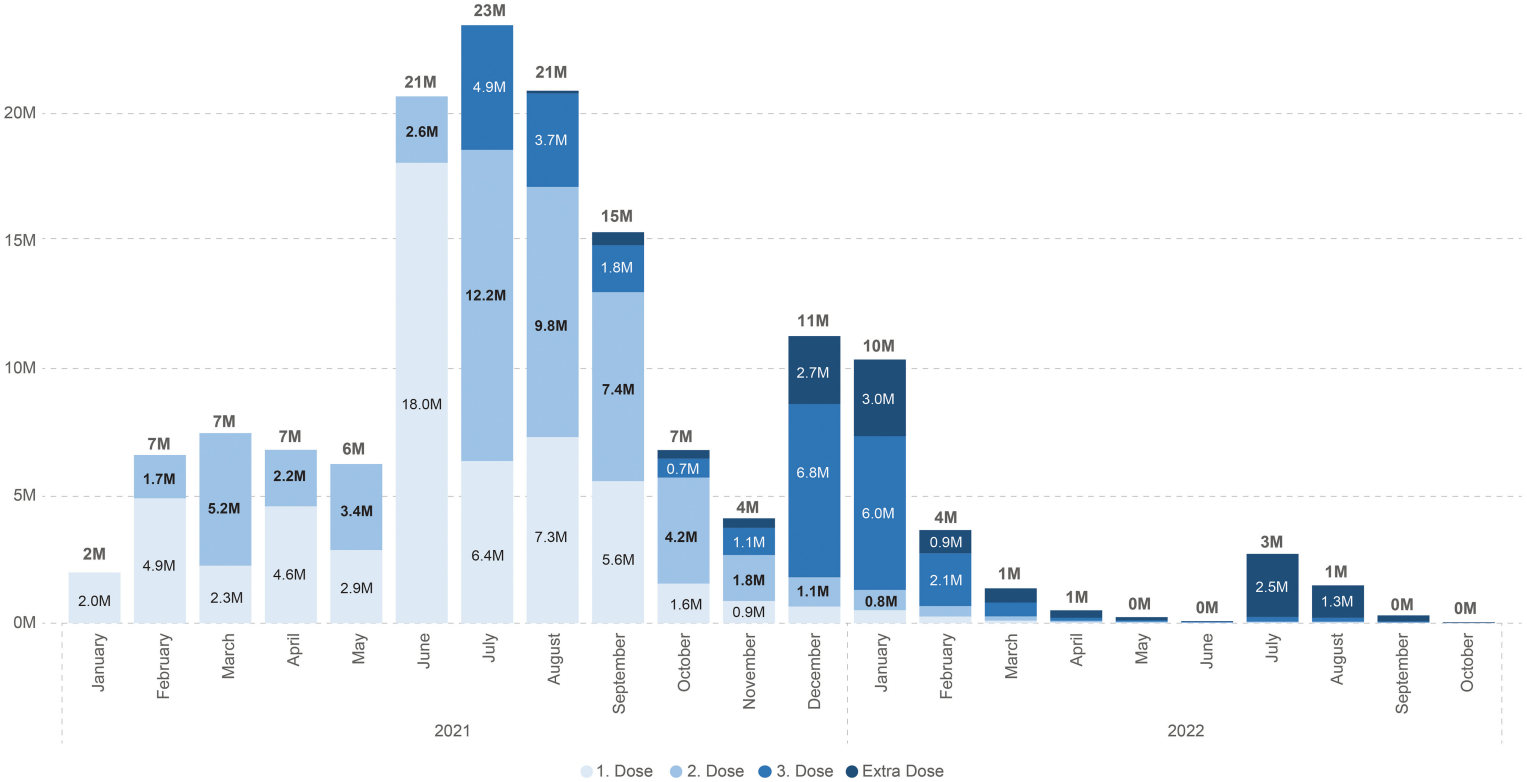
COVID-19 vaccination by month (graphic Zeki Berk, Türkiye).

Although Türkiye had an excellent infrastructure and system for vaccination programs, what started with good coverage faded with time (Figures 2, 3).

The Minister claimed that Türkiye would not need any imported vaccines from April 2021 because of the local domestic production of Türkiye's own COVID-19 vaccine, Turkovac (inactive type COVID-19 vaccine). However, Turkovac was authorized for emergency use only on December 22, 2021 (57). To-date, no results of the clinical studies on Turkovac were published or shared.

#### Risk communication and community engagement

Since all epidemics begin and end in communities, “risk communication and community engagement” (RCCE) is an essential element of emergency preparedness and response plan.

**Figure 4:**
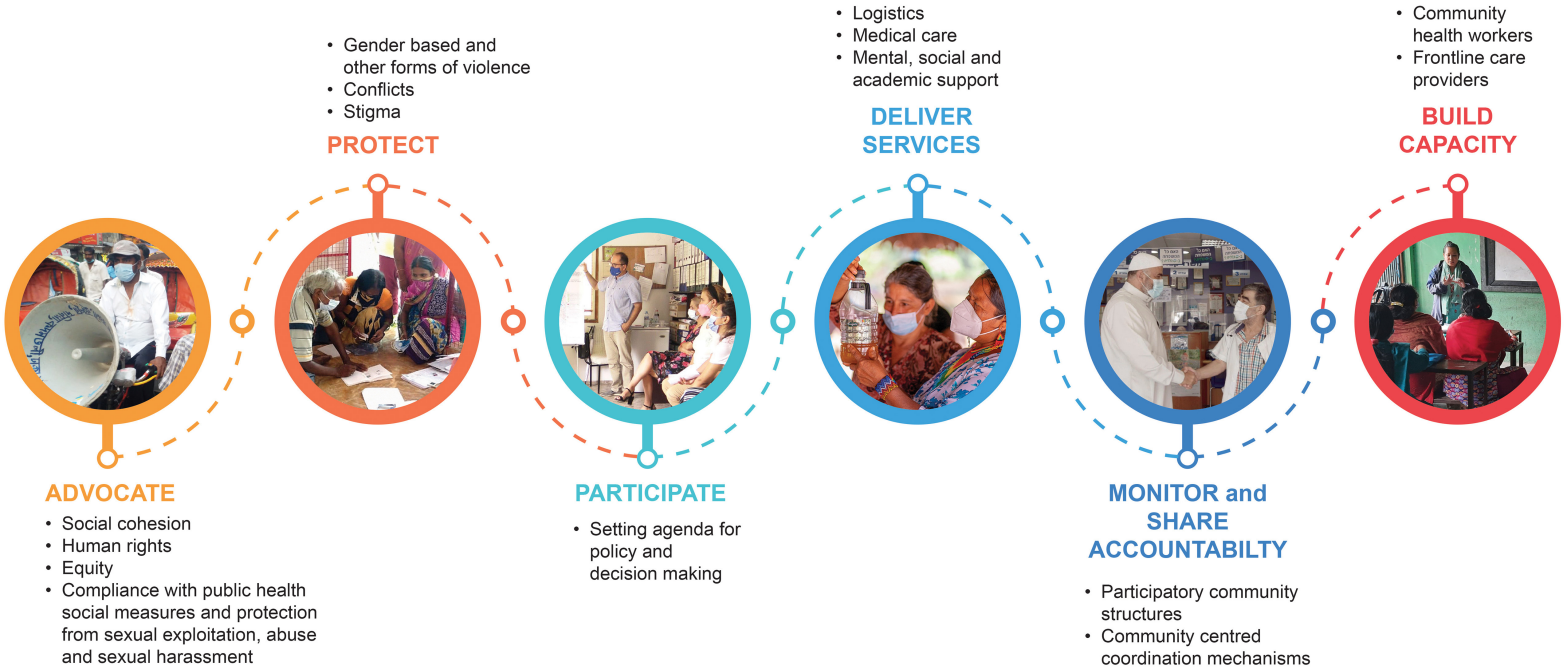
Civil society organizations' roles in mitigating COVID-19 impact at the community level (graphic Nellie Kartoglu/WHO).

Civil society organizations (CSO) play a critical part in supporting efforts in mitigating the economic, social, and health related harms of emergencies. Their role is also to expand, advocate, protect, participate, deliver services, monitor, share accountability, and enhance and expand capacity (Figure 4). CSOs, along with non-governmental organizations and professional associations, can be assets for government partners with their expertise in certain fields and their policy development skills, community outreach, and analysis. Especially in crisis situations like the COVID-19 pandemic, civil societies play a significant role in raising awareness on social inequalities that are deepened because of emergency situations (58).

From the beginning of the COVID-19 pandemic in Türkiye, the Ministry of Health acted as the sole authority by refusing to collaborate with the CSOs and professional organizations like the TMA. The TMA, despite continuous attempts, failed to secure an appointment with the Ministry of Health throughout the pandemic. On September 2, 2020, the deputy Minister of Health called a TMA COVID-19 Monitoring Board member to say that the Minister wanted to see him along with the TMA President the following day, this meeting took place with no outcome (59).

There is no indication of the Ministry having a RCCE plan. Throughout the pandemic, communication with the public was mainly through sporadic press releases and social media messages (Twitter especially) from the Minister. The Ministry was always reluctant in sharing data. Refusal to collaborate and non-transparent policies by the Ministry of Health brought frustration to the scientific community. This frustration reached its peak when the Ministry announced a mandatory application for permission to conduct research related to COVID-19, before any application to ethics committees. Consequently, the Ministry rejected some research applications with no explanation (60). This control was ended only on April 28, 2022 (61).

According to WHO, the COVID-19 pandemic was accompanied by an unprecedented infodemic (62). As too much information, including false or misleading information in digital and physical environments, started to circulate, conspiracy theorists, along with anti-vaccine groups, showed a strong presence on social media. In Türkiye, these groups organized demonstrations and painted vocal scientists as targets, reaching the degree of assault in certain cases (63). Despite death threats to eminent scientists, the Ministry of Health decided to remain silent. As indicated in an OECD study from 67 government evaluations (64), “trust requires transparency, not only through frequent and targeted crisis communication, but, more importantly, by engaging stakeholders and the public in risk-related decision-making.” In this regard, it can be concluded that trust cannot be built without a whole-of-society approach, where governments engage all stakeholders including the civil society, communities, academia, media, private sector, non-governmental organizations, other voluntary associations, families, and individuals to strengthen the resilience of communities and society as a whole.

### Assessment of effectiveness of control measures

Systematic surveillance and reporting are paramount in the control of epidemics and, more notably, in evaluating the effect of the control measures in use. These need to be communicated not only to decision makers but also to communities with full epidemiological details such as age, gender, occupation, place of residence, and social class. To contain the epidemic, it is essential to plan effective control strategies and forecast using epidemiological models to assess the course of the epidemic. For human-to-human transmission, the SIR (susceptible, infected, and recovered) model is widely used (65). More comprehensive models such as the SEIR (susceptible, exposed, infectious, removed) have also been developed for the COVID-19 pandemic (66, 67). However, there has not been an approach by the Ministry of Health in Türkiye in which the effect of epidemic control measures has been systematically evaluated using an epidemiological model. The Ministry of Health has not published any official reports containing the change in epidemiological indicators. Apart from the total number of confirmed cases and the total number of confirmed deaths due to COVID-19, no further details have been disclosed. As a result, independent scientists have not had the means to assess the impact of epidemic control measures.

## Recovery

Resilient recovery efforts following outbreaks like COVID-19 are critical both economically and socially as part of the continuum of the response cycle to emergencies (12). Since the pandemic affected all sectors, the policy choices governments will make today will determine the success of how we will be prepared for future threats. If we take education as an example, we should ask ourselves whether our government is coming up with a plan to upgrade schools' infrastructure and information technology know-how, and support teachers. To-date, no recovery plans have been announced in Türkiye.

## Actionable recommendations

Pandemic preparedness was insufficient globally. In addition, there has not been a strong response to the COVID-19 pandemic in Türkiye due to the inadequacy of the health system, the inappropriateness of the chosen strategies, and poor management. Based on the evaluation of the current pandemic response in Türkiye, if major policy changes are not introduced, we believe the next pandemic will hit even harder.

Authors considered the following facts in formulating the actionable recommendations for a better preparedness and response to future pandemics within the strategic plan as described by the WHO (23).

First, it is important to recognize the complexity and dynamic nature of pandemics, and to acknowledge that effective management will require ongoing adaptation and flexibility.

Second, within the whole-of-society approach, it is important to engage a diverse range of stakeholders in the management process, including public health officials, policymakers, healthcare providers, researchers, community leaders as well as CSOs. This can help to ensure that a variety of perspectives are taken into account, and that solutions are tailored to meet the needs of different populations and communities.

Third, it is important to take a multidisciplinary approach to pandemic management, recognizing that pandemics are not just a medical issue, but also have social, economic, and political dimensions. This may involve collaboration across different sectors and disciplines, such as public health, economics, and sociology.

Fourth, it is important to recognize that pandemic management will involve trade-offs between different objectives, such as protecting public health while minimizing economic disruption. These trade-offs will need to be carefully considered and balanced to ensure that the most effective solutions are found.

Finally, it is important to continuously monitor and evaluate the effectiveness of pandemic management strategies, and to adapt and refine them as needed based on new data and insights.

### Coordination, planning, financing, and monitoring

1. A thorough and detailed pandemic management evaluation should be conducted involving all sectors affected by the pandemic.
2. The national pandemic preparedness and response plan should be updated based on the outcome of this evaluation and the plan should be integrated into national emergency preparedness framework.
3. Whole-of-society approach should be supported, and multi-stake holder and multi-sectoral coordination between ministry of health and public health authorities and non-health sectors should be strengthened and respected throughout the pandemic.
4. The scientific advisory board should consist of scientists who are competent in the field and should be inclusive of key-players in an emergency setting. The decisions of the Board should be announced to the public by the spokesperson of the Board. The Ministry of Health should only attend scientific advisory board meetings as an observer, not to chair.
5. Within command and control, transparent national norms should be followed, and confusing involvement of other ministerial bodies should be avoided.
6. Türkiye should increase significantly its budget devoted to pandemic preparedness and response. In addition, the portion of the gross national product (GDP) marked for health expenditures should also be brought to at least 8% of GDP.
7. The government should provide real time information on resource mapping and expenditure tracking to allow equitable access to resources and as well as for transparency and accountability.
8. Trust should be maintained across all agencies and organizations and with the public through a commitment to transparency and credible actions.

### Risk communication, community engagement, and infodemic management

9. A risk communication and community engagement strategy should be developed.
10. Effective dialogue and listening mechanisms with the general public should be established.
11. Communities, professional societies and relevant CSOs should be empowered and be engaged in the production, validation, and dissemination of information as part of the whole-of-society approach.
12. A rumor tracking system should be set up to closely monitor misinformation and disinformation with respond mechanisms to prevent and to mitigate its impact on health of population. Legal arrangements should also be set to combat disinformation.
13. Individuals and communities should be provided with timely, detailed, and reliable information online and offline throughout the pandemic.

### Surveillance, epidemiological investigation, contact tracing, and adjustment of public health and social measures

14. National surveillance system should be strengthened to collect up-to-date clinical, virological, and epidemiological information to rapidly detect, investigate and report new cases and clusters.
15. Public health and social measures should be implemented as part of risk mitigation strategies that are multi-layered, science based and subjected to effectiveness checks and formal reviews.
16. Evidential basis should be used for targeted interventions, such as public health intelligence, health system capacity, utilization of facilities, community risk factors and vulnerabilities.
17. Sequencing of viruses within surveillance activities should be increased.
18. Data stratified by basic descriptive epidemiological elements should be compiled, analyzed. Epidemiological situation as well as the responses by the health system should be transparently and timely shared with the public.

### Points of entry, international travel and transport, and mass gatherings

19. Pandemic related international border closures for people and cargo should not be enforced.
20. Restrictions of in-country travel should not be enforced unless participating in a globally led containment operation.
21. Restrictive measures of mass gatherings should be implemented equally to all sectors and should not be waived in favor of ruling political power. Results of the mass gathering risk assessments should clearly be communicated to public.

### Laboratories and diagnostics

22. National case definitions should be in line with WHO guidelines and ICD-10 coding. Clinical and laboratory algorithms should also be updated to reflect international harmonization.
23. Laboratory diagnostic capacities should be enhanced and expanded to characterize virus isolates and related information using protocols and procedures developed in collaboration with WHO.
24. In addition to PCR testing, rapid antigen-detection tests (Ag-RDTs) tests should be made available to public.

### Infection prevention and control, and protection of the health workforce

25. Infection prevention and control measures in health facilities should be endorsed with consistent approach to guidelines, training, implementation, and monitoring.
26. Adequate patient to health workforce ratio should be achieved.
27. Health workforce should be supported with access to supplies and personal protective equipment (PPE). Procurement and distribution of PPE for protection of health workforce should be planned.

### Case management, clinical operations, and therapeutics

28. Case management guidelines should be in line with internationally accepted WHO recommendations.
29. Science-based mechanisms and procedures should be developed to select, procure, stockpile and distribute therapeutics.

### Operational support and logistics, and supply chains

30. Operational and logistics response teams should be established to tackle mobilizing and dispatch resources for rapid containment.
31. Stockpiles should be planned for diagnostics, PPE, antivirals, and vaccines. Stockpiles of pharmaceuticals and other materials should be distributed according to established national plans.

### Maintaining essential health services and systems

32. Health investments should be strengthened to support essential health services during emergencies. Financial barriers to access should be removed.
33. Regular monitoring should be conducted to assess service availability, barriers to access, and use of essential health services to allow priority decisions.
34. A designated focal point should be appointed for essential health services as the member of scientific advisory board during pandemics.
35. Health workforce capacity should be rapidly optimized in support of essential health services.
36. Communication strategies should be strengthened to support the appropriate use of essential health services.

### Vaccinations

37. The selection of vaccines for procurement should be communicated to public and health professionals transparently.
38. Effective vaccines should be made available for all population with no access problems.
39. Conduct a thorough evaluation of all pharmaceutical interventions including vaccine coverage, effectiveness, and safety.
40. Effective strategies should be established to combat misinformation and disinformation on vaccinations.

## Discussion

Within the above-mentioned actionable recommendations, several areas must be underlined for their crucial role in the success on preparedness and response to future pandemics. These points mentioned below are in line with findings of Turkish Medical Association that are published in its several reports throughout the pandemic (20, 21, 41).

A whole-of-society approach in preparedness and response is paramount in building trust, therefore success. Inclusiveness should be practiced at all phases of pandemic continuum. In this regard, a thorough evaluation of the pandemic management involving all sectors affected by the pandemic would be an essential step.

The current 3.6% of budgetary provisions for health services should be increased to at least 8% share of the GDP, as is the case in countries with similar GDP (68). This increase should include support to essential health services.

Development and implementation of a risk communication and community engagement strategy is critical in practicing a clear and transparent communication with the public. As was the case during the COVID-19 pandemic, no information should be withheld from the community. Access to information on a timely manner is a basic human right and belongs to everyone.

There were examples of government lifting the mass gathering bans only for periods to facilitate governing party manifestations and then enforcing the ban again. Scientific reasoning must be exercised in all decisions without any partisan motives.

Testing policies, treatment guidelines, and vaccination policies were behind the acceptable norms and priority should be given to update them with international accepted science-based policies.

It is also critical that Türkiye should update and revise the very old Law on Public Health (No. 1593) enacted in 1930 which has been amended several times since then. Despite it continues to provide a general legal framework for public health including response to infectious diseases and pandemics, some provisions are outdated, and it should be reframed fully with all new public health approaches.

The authors would like to conclude this evaluation with a quote from the Global Health Center, Graduate Institute of International and Development Studies based in Geneva, Switzerland (69).

“COVID-19 has provided a stark reminder, not only that many wealthy nations suffer from significant weaknesses in national preparedness, but also that international arrangements are patchy, weak, and wholly inadequate in scope and strength. Without far-reaching reforms at national and global levels, future crises will hit hard. However, it is likely that the post-COVID reform process will suffer from its own blindspots and only a few major reforms will likely be implemented. The selection of those reforms will not be a purely technocratic process, but a political one. Therefore, it is critical that arrangements for robust monitoring, and regular revision of existing arrangements are put in place, that will allow for periodic reforms to the system as we inevitably switch from fighting the last war to confronting new outbreaks.”

## Author contributions

All authors listed have made a substantial, direct, and intellectual contribution to the work and approved it for publication.
